# Identifying specular highlights: Insights from deep learning

**DOI:** 10.1167/jov.22.7.6

**Published:** 2022-06-17

**Authors:** Eugen Prokott, Roland W. Fleming

**Affiliations:** 1 Department of Experimental Psychology, Justus-Liebig-University Giessen, Giessen, Germany; 2 Department of Experimental Psychology, Justus-Liebig-University Giessen, Giessen, Germany; 3 Center for Mind, Brain and Behavior, University of Marburg and Justus-Liebig-University Giessen, Giessen, Germany

**Keywords:** material perception, intrinsic image analysis, gloss, reflectance, specularity, inverse optics, surface

## Abstract

Specular highlights are the most important image feature for surface gloss perception. Yet, recognizing whether a bright patch in an image is due to specular reflection or some other cause (e.g., texture marking) is challenging, and it remains unclear how the visual system reliably identifies highlights. There is currently no image-computable model that emulates human highlight identification, so here we sought to develop a neural network that reproduces observers’ characteristic successes and failures. We rendered 179,085 images of glossy, undulating, textured surfaces. Given such images as input, a feedforward convolutional neural network was trained to output an image containing only the specular reflectance component. Participants viewed such images and reported whether or not specific pixels were highlights. The queried pixels were carefully selected to distinguish between ground truth and a simple thresholding of image intensity. The neural network outperformed the simple thresholding model—and ground truth—at predicting human responses. We then used a genetic algorithm to selectively delete connections within the neural network to identify variants of the network that approximated human judgments even more closely. The best resulting network shared 68% of the variance with human judgments—more than the unpruned network. As a first step toward interpreting the network, we then used representational similarity analysis to compare its inner representations to a wide variety of hand-engineered image features. We find that the network learns representations that are similar not only to directly image-computable predictors but also to more complex predictors such as intrinsic or geometric factors, as well as some indications of photo-geometrical constraints learned by the network. However, our network fails to replicate human response patterns to violations of photo-geometric constraints (rotated highlights) as described by other authors.

## Introduction

Humans easily perceive and distinguish materials visually. One important optical aspect of materials is glossiness. It is useful in determining whether a piece of food is fresh, whether the floor is slippery, or whether a surface is clean or greasy. Arguably the most important image feature for gloss perception is the presence of highlights (Beck & Prazdny, 1981; Fleming, 2012; Marlow, Kim, & Anderson, 2012)—direct reflections of light sources or other bright elements in the environment.

The exact computations underlying human perception of highlights and gloss remain poorly understood. Factors other than the physical reflectance properties of a material itself, such as shape or illumination, can impact perceived gloss (Doerschner, Boyaci, & Maloney, 2010; Fleming, 2012; Fleming, Dror, & Adelson, 2003; Ho, Landy, & Maloney, 2008; Olkkonen & Brainard, 2011; Wijntjes & Pont, 2010). This has been taken to indicate that the human visual system does not accurately estimate the physical reflectance of surfaces but rather arrives at a subjective impression of gloss through the use of heuristics, in which properties of highlights, such as shape, contrast and size play an important role (Fleming, 2012; Marlow et al., 2012). The importance of highlights is well known (Beck & Prazdny, 1981; Todd, Norman, & Mingolla, 2004), but it remains unclear what exact computations the visual system uses to recognize highlights—that is, to distinguish specular highlights from other bright patches in the image, such as texture markings. The perception of materials and glossiness is typically associated with mid-level vision (Anderson, 2011; Fleming, 2014; Komatsu & Goda, 2018) in which low-level image features such as edge orientation, color, brightness and gradients, or scale are pooled and compared to arrive at surface representations. Several studies have shown the importance of three-dimensional (3D) surface representations and the need for specular highlights to be congruent with surface geometry and shading patterns to elicit a perception of gloss (Anderson & Kim, 2009; Beck & Prazdny, 1981; Kim & Anderson, 2010; Kim, Marlow, & Anderson, 2011; Marlow, Kim, & Anderson, 2011; Todd et al., 2004). Anderson and Kim (2009) showed that images in which the highlight component has been rotated with respect to the matte component are less likely to be perceived as glossy. Marlow, Todorović, and Anderson (2015) and Marlow and Anderson (2015) demonstrated how identical image gradients can be interpreted as blurry highlights on a glossy surface or shading on a matte surface depending on perceived 3D surface structure. Yet, despite progress in determining many of the factors that influence the identification and interpretation of highlights, there is still no image-computable model that emulates human judgments.

To address this need, in this study we took a big data approach to modeling highlight perception. Specifically, we sought to develop a model that distinguishes whether bright markings in images of surfaces appear as highlights rather than texture or some other non-highlight feature. We used machine learning as a method that allows us to train a model on thousands of images with random variations in geometry, texture, and illumination to capture those features that are useful for identifying highlights over a wide range of surfaces and appearances.

This is essentially an “intrinsic image decomposition” task—a well-known problem in the computer vision literature that has been studied since the late 1970s (Barrow & Tenenbaum, 1978; for a recent review, see Bonneel, Kovacs, Paris, & Bala, 2017). Most computational models on highlight detection, however, focus on removing highlights, as they interfere with identifying other intrinsic components such as shading or surface reflectance. Here, we focus not on a full decomposition into intrinsic components but specifically on isolating the specular component of images. Importantly, rather than solving the engineering goal of identifying highlights as accurately as possible, we focus on reproducing the pattern of behavior that humans exhibit— both successes and failures.

Previous work comparing convolutional neural networks (CNNs) to humans has shown both striking similarities (Gomez-Villa, Martin, Vazquez-Corral, & Bertalmio, 2018; Ward, 2019; Watanabe, Kitaoka, Sakamoto, Yasugi, & Tanaka, 2018) and also discrepancies where CNNs react very differently from humans to slight changes in a stimulus (Kurakin, Goodfellow, & Bengio, 2019; Nguyen, Yosinski, & Clune, 2015; Sharif, Bhagavatula, Bauer, & Reiter, 2016; Szegedy et al., 2014), show different generalization behavior to humans (Nguyen et al., 2015), or have difficulties solving visual tasks that are very simple for humans (Stabinger, Rodríguez-Sánchez, & Piater, 2016). Networks are often susceptible to being fooled by specific small changes that are almost imperceptible to humans called adversarial attacks (Szegedy et al., 2014), and the performance of a CNN often decreases catastrophically when confronted with degraded stimuli (Geirhos, Temme, Rauber, Schütt, Bethge, & Wichmann, 2018), unlike humans. In addition to picking up on pixel artifacts, there are also reports of CNNs learning different cues and mechanisms than humans. For example, CNNs have been found to make different use of scene context than human observers, outperforming humans in recognizing objects from their backgrounds only (Zhu, Xie, & Yuille, 2017). Geirhos, Rubisch, Michaelis, Bethge, Wichmann, and Brendel (2018) found that CNNs trained on the ImageNet classification task (Russakovsky et al., 2015) tend to rely heavily on texture rather than object shape.

As one approach to mitigating these tendencies, here we use pruning as a method for fine-tuning a trained neural network to make it respond more similarly to humans. Although many other approaches are possible, pruning is straightforward and does not require enough human data to train a network from scratch. It has been used for over three decades as a method for reducing network complexity and computational requirements (Janowsky, 1989; LeCun, Denker, & Solla, 1990; Mozer & Smolensky, 1989). In simple terms, the rationale for pruning is that a complex network will typically contain both necessary and unnecessary units. Identifying and pruning unnecessary units can reduce network complexity while retaining high performance. There is usually a trade-off between network performance and reducing network complexity. The exact criteria for evaluating the importance of single units and the pruned network overall are a subject of much research (for a recent review, see Blalock, Gonzalez Ortiz, Frankle, & Guttag, 2020). It has been observed that pruning can improve generalization performance (Bartoldson, Morcos, Barbu, & Erlebacher, 2020; Hassibi & Stork, 1993; LeCun et al., 1990) and in some cases that pruned networks outperform the original unpruned networks (Han, Pool, Tran, & Dally, 2015; Suzuki et al., 2020) on the training objective.

Yet, here, we use pruning as a method for optimizing the network functionality not in terms of the original training criterion but rather *human responses* on the same task. We expect that a neural network trained to identify physically accurate highlights will learn an approximate solution that includes features similar to those the human visual system uses but also includes different features. We therefore hypothesized that in a second fitting stage we can prune the trained network to identify a variant that emphasizes the similarities to human responses while de-emphasizing the differences. Given this pruned network, we can also test whether it is possible to gain insights into the processes that make the model respond similarly to human observers.

To do this, we created a large dataset of 164,085 images containing glossy surfaces with varying textures or without texture. To limit the number of human responses required, we investigated the similarities in predictions only in certain pixels that we expected to be particularly informative. To identify these pixels, we used two extreme predictions as baseline models. One is a very simple model that we trained to learn a global intensity threshold value for classifying brighter points as highlights. Although crude, such a heuristic can correctly identify highlights in many conditions. However, as it lacks any knowledge of surface or image geometry, it can also be readily fooled by bright texture markings. The other extreme was the physical ground truth from our rendering simulations, representing a physically correct solution—the upper limit on performance that any observer system could achieve. We expect the human visual system to make more sophisticated decisions than an intensity threshold but also simpler inferences than a fully accurate inverse physics estimate. We therefore expect to find informative image locations that are particularly descriptive of human highlight perception where our two baseline models contradict each other. We chose image locations based on these two predictors, including some where both models agree, to check whether our stimuli and experimental method yield meaningful perceptual responses in conditions where we have a clear expectation of subjects’ responses. We ran two parallel experiments, the stimuli being constructed and chosen according to the same principles but chosen from a different set of images.

To anticipate the main results as a basis for outlining the modeling approach, we find that observers respond to pixels where both models agree mostly in line with model predictions. For the two disagreement categories, subjects’ responses are mixed, and neither ground truth nor the threshold model better predicts human highlight perception. This provides us with a promising basis to develop a better model of human perception.

We trained a novel CNN architecture (see Methods) to predict for each point in the image whether or not it contains a highlight, using supervised training with the ground truth rendered specular component of the images as a label for each pixel. This model predicted human responses on the probe locations better than ground truth or the threshold model. To further fit our model to human responses we pruned network connections using a genetic algorithm in order to identify those configurations of pruning that maximize correlations to humans on the target dataset. A genetic algorithm allows us to test model fitness of various configurations of several connections being pruned at the same time.

We find that indeed a large set of pruned configurations correlate higher with human responses than the full network. We examined one representative subnet in detail, investigating where the differences to the full network lie. We also conducted a representational similarity analysis (RSA) (Kriegeskorte, Mur, & Bandettini, 2008) to compare intermediate layers of the pruned network to various candidate predictors. We find that representations within the network are similar to various simple and more complex predictors suggested in human gloss perception literature. We also demonstrate that our network is weakly sensitive to violations of photo-geometrical constraints of highlights. In a lesion analysis, however, we find no evidence that neurons with high similarity to geometric predictors are more important than other neurons for the model to react like human observers.

Our main result is that we have developed, to the best of our knowledge, the first image-computable model that predicts human highlight judgments better than ground truth. Although the network learned representations related to photo-geometrical predictors, surprisingly we find that these features are no more important than simpler image-computable features for the model to respond similarly to humans. Our findings also show an application of pruning neural networks based on a relatively small human dataset as a method for fine-tuning neural networks that were previously trained on simulated physical data. This method could be beneficial in other research areas where machine learning would be useful, but target data are slow or costly to obtain and simulated data are more readily available.

## Methods

### Training data and stimuli for experiment with human observers

**Figure 1.**
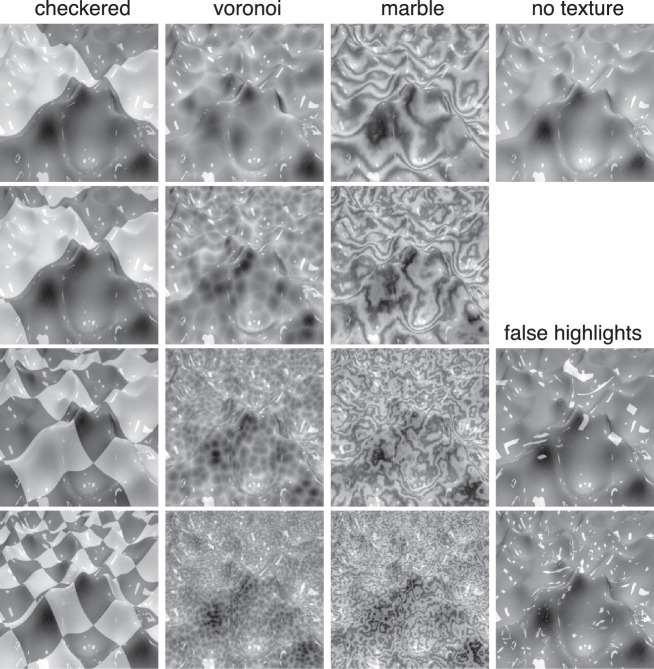
Example images showing all different texture conditions for one scene.

To train and test our networks and to test human observers we rendered 164,085 grayscale images of glossy surfaces like those shown in Figure 1. The image size was 256 × 256 pixels. Every image was filled by a surface viewed at 45° and perturbed by waves and illuminated by a square light source parallel to and at a random position above the surface. The geometry was generated using the Ocean Modifier in Blender. The light source was located near the surface, between 3.8 and 4.8 times the vertical distance between the camera and the surface. We used a simple illumination environment with a single light source before a dark background to make sure it is well defined which pixels contain specular highlights. Images were rendered in Blender using the Cycles render engine. We used four spatial scales of surface geometry. For every surface geometry, we rendered a plain surface consisting of an untextured specular and diffuse component combined, as well as 12 texture conditions (four spatial scales each of Voronoi patterns, marble patterns, and checker patterns). These textures were created as procedural textures in Blender using the Voronoi, Wave, and Checker nodes, respectively, and applied to the surface using UV mapping. In order to create texture patterns that could reasonably be confused with the highlights, we also rendered two versions of each scene where we used the specular reflections from a randomly chosen different surface as a texture map by multiplying it with the diffuse component (see examples in Figure 1, under “false highlights”). We chose these conditions to include textures that consist of highlight-like patches in positions and orientations that are incongruent with the surface, as Anderson and Kim (2009) have shown that matte surfaces with physically correct highlights imposed at a wrong orientation or position are less likely to be perceived as glossy. Our intention was that a network trained on these stimuli would be forced to learn something about highlight positioning (rather than just intensity), possibly yielding a wider range of strategies that could show up during the pruning stage of our network fitting.

Of the rendered images, 7200 were withheld from network training, balanced for surface scale and patterns, leaving 156,885 training images. The withheld images were used as candidates in selecting images and probe locations for the first human experiment and as a validation set during training. We also rendered a second set of 15,000 images constructed the same way as the initial dataset. These images were also not shown during training. They were used to select the images and probe locations for the second human experiment and as stimuli for additional network analysis.

### Experiment with human observers

**Figure 2.**
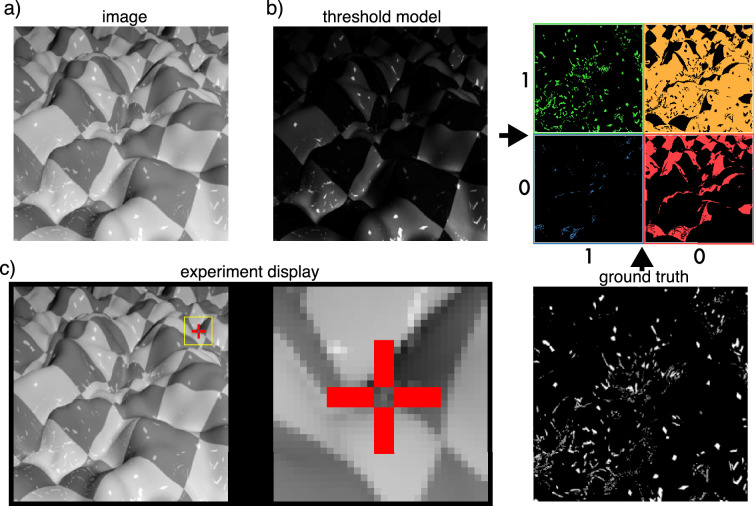
(a) An example image as shown to the network and observers. (b, left) Predictions by a simple threshold model (TM) of the image in (a). (b, bottom right) Ground-truth specular component (GT) used as labels during network training. (b, top right) The four categories from which probe locations for experiments with human observers were chosen. Green: TM correctly identifies a highlight at these locations. Yellow: TM wrongly identifies a highlight. Blue: TM fails to identify a highlight. Red: TM correctly identifies no highlight. (c) Example of the display seen by observers during the experiment. Subjects saw an image with one pixel marked by a red cross and a magnified close up (8×) of a 32 × 32 pixel patch that they could move with the mouse. The location mark could be toggled on and off.

Human observers were asked to judge specific pixels in an image and to respond whether or not the pixel contained a highlight. To make these pixelwise responses more informative, we did not select the locations at random (also because only about 3% of all pixels contained highlights). To select probe locations (see Figure 2 for an example), we trained a simple model that used a single global intensity threshold to identify highlights and obtained a prediction for each of 7200 test images. We also looked at the specular map (ground truth) for these images. From these two response maps we could sort each pixel into one of four categories: (a) both ground truth and the threshold predictor agree there is a highlight, (b) threshold predicts a highlight but there is no highlight according to ground truth, (c) there is a highlight but threshold does not predict one, and (d) threshold and ground truth agree that there is no highlight. Note that both the ground-truth specular map and the threshold predictor contained continuous information about the strength of specularity (i.e., the image intensity of specularly reflected light) or the strength of the estimate. The intensity varies, for example, at the edges of highlights and is thus a continuous quantity. To categorize pixels, we treated the predictions as binary (i.e., measuring whether there is any specularity in the prediction). For selecting single pixels from these categories, we used the original continuous information to maximize the function of each category. For each category we chose one pixel per image that maximized this function; for example, for category (b), we chose that pixel per image where the threshold model gave the highest prediction, although there was no highlight according to ground truth. The ground truth and threshold prediction are described in more detail in the next section. For 120 images we chose one probe for each of the four categories, and for another 120 images we chose one probe only for categories (b) and (c) where threshold and ground truth disagreed. These are likely to be more informative in discriminating between predictors than pixels where a simple model already agrees with ground truth. Pixels from categories (a) and (d) provide a useful baseline to determine whether our method of single-pixel judgments yields expected results for easy stimulus conditions and to confirm that observers perceive highlights in our stimuli as such.

We constructed two test sets. For the first—which we refer to as the *target set—*the images were chosen randomly from a pool of 7200 candidate images. For the second test set, which we used as a *validation set*, images were chosen from a pool of 15,000 images. For both sets, images were chosen in such a way as to balance surface scale and texture conditions. In both cases, we tested subjects on 720 probe locations on 240 images, showing images in random order. Each trial consisted of a display of the image, with the current probe location marked by a red cross. Next to the image was a second display of a close-up of a 32 × 32-pixel image patch, magnified 8×. Figure 2 shows an example display. By default, this was centered on the probe location but could be moved using the mouse. The cross indicating the probe pixel could be toggled on and off. Subjects were asked to judge the central pixel and respond with one of two keys whether this pixel contained a highlight/reflection or texture.

Thirteen participants 20 to 39 years of age (mean, 25.9) took part in the first experiment. In the second experiment, 15 observers 19 to 33 years of age (mean, 25.0) participated. They all had normal or corrected-to-normal vision and signed informed consent according to the tenets of the Declaration of Helsinki. The procedures were consistent with those approved by the local ethics committee of the Psychology Department at the Justus-Liebig-University of Giessen. Both experiments lasted about an hour.

### Ground-truth and threshold model predictions

The ground-truth information used for choosing probe locations for the experiments with human observers was obtained as a rendering of the untextured surface in a material reflecting only specularly in Blender. Note that these ground-truth maps are not binary, although they may look so, and contain variation in specular intensity. For the threshold predictor, we trained a model to determine a global threshold for the 156,885 images that we also used for training the neural network. This model was implemented in TensorFlow as a neural network with one convolutional layer consisting of a single pixel filter and a ReLU output. A second layer with a sigmoid activation function mapped the activations into the displayable intensity range. This way the model prediction was only based on the individual intensity of each pixel, regardless of neighbors and context. The model contained a threshold below which predictions were 0 and otherwise gave a continuous prediction between 0 and 1. We trained the model for 50 epochs using binary cross entropy as the objective loss function. This results in near-optimal threshold for approximating the ground truth.

To categorize candidate pixels into categories (a) to (d) described earlier, we used binary data from each predictor (whether a predictor gave a zero or non-zero prediction to each pixel). To choose pixels within each category we used the continuous prediction values to maximize the function for each category. For example, for category (b), where the threshold predicts a highlight where there is none, we picked out of all candidate pixels in an image the pixel for which the threshold model gave the highest prediction. For an example of the model outputs and the probe location selection see Figure 2.

### Network architecture

**Figure 3.**
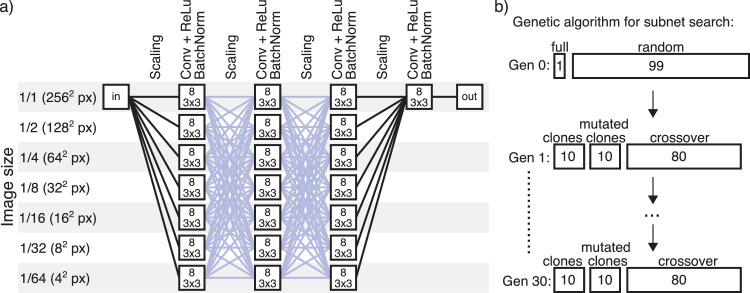
(a) Network architecture. The first three tiers (columns) consist of seven parallel convolutional layers (boxes), each processing at different resolutions from full resolution to 1/64 resolution. Each convolutional layer contains eight filters (filter size is 3 × 3 pixels). The connections shown in blue are the ones that were subject to the pruning search. (b) A schematic of the search algorithm. The initial generation consisted of the full network and 99 random subnets. Each following generation consisted of 10 clones of the fittest networks of the previous generation, one mutated version of each of these 10, and 80 subnets created through crossover from two members of the previous generation, picked according to fitness. Fitness was defined as the correlation to humans on the target set.

The network architecture we used is shown in Figure 3a. It was designed to give the network capabilities of performing image computations at different spatial scales and to exchange processing results between scales between layers. The network consists of four tiers of parallel convolutional layers. We refer to these as “tiers” to avoid confusion with “layers,” as they are implemented in deep learning software. The first three of these tiers consist of seven parallel convolutional layers each. These parallel layers each receive the same input at different scales ranging from 1/1 size to 1/64 size. Each convolutional layer contains eight filters. Before the first tier of convolutional layers, the input image is scaled to these seven different sizes. After convolutional processing, there are again scaling layers so that the output of all layers operating at different scales is scaled up and/or down in order that each of the parallel layers in the next tier receives input from each of the parallel layers in the previous tier. This scaling happens again between the second and third tier. The fourth tier consists of one layer with eight convolutional filters at full image scale, after which comes an output layer of one channel.

### Network training

We performed feedforward training on our network, using the ground-truth specular maps as labels and with binary cross-entropy as our loss criterion. Training lasted for 50 epochs; that is, the training algorithm circled through the entire training image set in randomized order 50 times.

### Network pruning

Pruning was performed on the trained network. Rather than pruning individual neurons, we deleted connections between the first, second, and third tiers of parallel layers, where each layer contained eight convolutional filters. Each of these tiers consists of seven parallel convolutional layers processing the image at different spatial scales, each of which feeds into each of seven parallel nodes in the subsequent layer. This amounts to 98 connections between the layers in the first and second and the second and third tiers.

To perform and evaluate pruning we used a genetic algorithm. Each network configuration can be described by a 98-parameter vector, where each parameter can either be a 1 (connection on) or a 0 (connection off). The “full network” refers to the network configuration vector of only 1s, where all connections are active. During training all connections were active. We started each run of the algorithm with a population of 99 random configurations (in which every connection had a 50% chance of being pruned) and the full network. We applied each configuration to the trained network, obtained predictions for the test images, and took the predictions for those pixels that we showed to humans in our experiment. From the response vector on these pixels, we calculated a correlation to humans and used it as a fitness criterion in the genetic algorithm. After calculating the fitness for an entire population (i.e., 100 configurations) we kept the 10 fittest members as survivors, added a mutated (5% chance of a switch between 0 and 1 for each connection) copy of each survivor to the next generation, and added 80 configurations through cross-over from members of the previous generation (chosen randomly, weighted by fitness, 1% mutation chance). We repeated this process for 30 generations and ran 300 instances of the genetic algorithm.

## Results

### Humans

Human observers were asked to judge whether or not 720 individual probe locations contained a highlight. These probe locations (single pixels) were picked based on ground truth and a threshold model that used only an intensity threshold to predict highlights. Figure 2 illustrates the probe location selection process.

**Figure 4.**
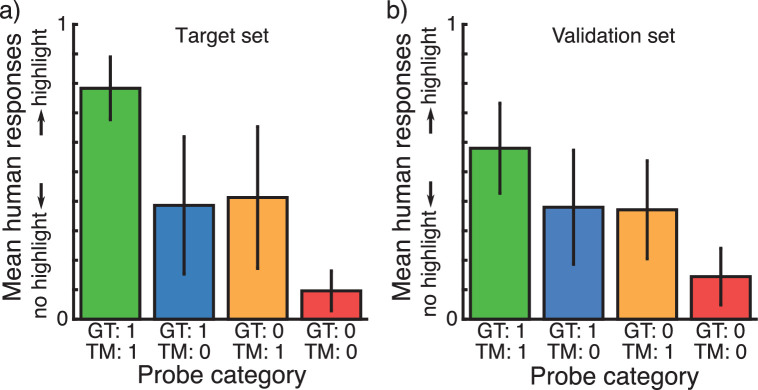
(a) Mean results from 13 human observers on the target set, one point from each category described in Figure 2b selected for each of 120 images. One point from only the blue and orange categories (disagreement categories) for another 120 images. (b) Mean results from 15 human observers on the validation set; a second set of probe locations and images constructed and selected according to the same criteria as those in the target set.

Mean responses from Experiment 1 grouped by the categories of probe locations are shown in Figure 4a. Results from the first experiment were later used as a target for our pruning algorithm and are referred to as the *target set*. Mean responses from Experiment 2 are shown in Figure 4b. The probe locations in this experiment were selected from a different set of images according to the same criteria as the target set. Results from the second experiment were used for validation and are referred to as the *validation set*. Note that the purpose of the validation set was not to validate human behavior in the target set, but rather to validate how well the model's imitation of human behavior generalizes to a new set of stimuli and locations. We therefore do not test for behavioral differences between the two human response sets.

Results show, as expected, that category (a) pixels, which contain highlights and are brighter than the threshold (see Methods), are most likely to be classified as highlights. Similarly, category (d) pixels, which do not have a highlight and are darker than the threshold, are least likely to be classified as highlights. Interestingly, pixels from categories (b) and (c), which either contain a highlight but are darker than the threshold or contain no highlight but are brighter than the threshold, are on average similarly likely to be judged as a highlight. This suggests that sheer relative pixel intensity does have an impact on human highlight perception, but that further factors play a role.

**Figure 5.**
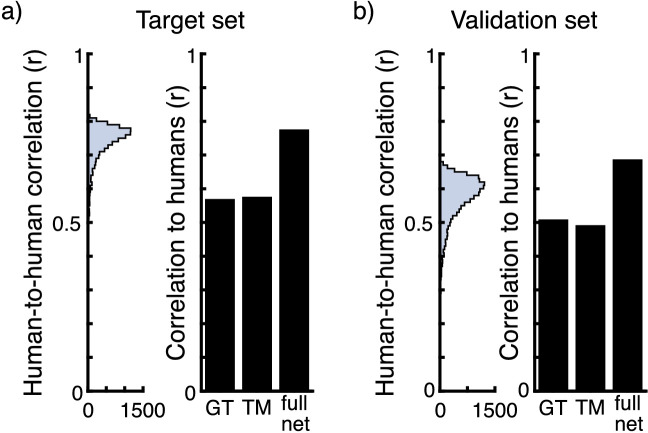
(a, left) Histogram of human-to-human correlations between responses to the target set calculated by randomly splitting the group of participants in two 10,000 times and correlating their mean responses. (a, right) Correlation of the threshold model (TM), ground truth (GT), and full network to the mean of human observers on the target set. (b, left) Histogram of human-to-human correlations between responses to the validation set calculated by randomly splitting the group of participants in two 10,000 times and correlating their mean responses. (b, right) Correlation of the TM, GT, and full network to the mean of human observers on the validation set.

The pattern of results for the four pixel categories is very similar for both experiments. It shows that human observers perceived highlights in our stimuli and that they were able to interpret and respond to single pixel probe locations. Both ground-truth and threshold predictions seem to partially predict mean human responses equally well (correlation to mean human responses *r* = 0.57 and *r* = 0.58 for the target dataset and *r* = 0.51 and *r* = 0.49 for the validation dataset, respectively). As a comparison we calculated the intercorrelation among human observers. Because human responses were binary, we randomly divided the observer group in two 10,000 times, correlating the mean responses of the two groups every time. The maximum correlations we observed were *r* = 0.82 for the target dataset and *r* = 0.69 for the validation dataset (mean correlations were *r* = 0.73 and *r* = 0.57, respectively). Figure 5 shows the distributions of these human-to-human correlations. A large proportion of the variance in human responses remains unexplained by other observers.

To test whether this is due to idiosyncratic response behavior or simply noisy responses, we compared inter-rater and intra-rater agreement. Because the responses of individual observers are binary, we could not calculate this as a correlation and chose to use the rate of agreement as a measure of consistency. To calculate inter-rater consistency, we defined comparable pixels according to the same pixel categories (a) to (d) (Figure 2), same image texture category, and same surface geometry scale. We split each group of comparable pixels in half randomly, thus splitting the entire target set in half with a comparable counterpart for each pixel in the two halves. We calculated intra-rater consistency as the rate of agreement between an individual's responses to comparable pixels in the two halves and inter-rater consistency as the rate of agreement between an individual's responses and other individual's responses to comparable pixels. We repeated this process 1000 times to get an estimate of the inter- and intra-rater consistency. A paired *t*-test of the per-subject means of these two consistency distributions showed a significant difference, *t*(12) = 6.62, *p* < 0.001, with intra-rater consistencies higher than inter-rater consistencies (mean ± *SD*, 0.71 ± 0.06 vs. 0.61 ± 0.07, respectively). As a measure of the effect size, Cohen's *d* = 1.84. The same analysis for the validation set responses also revealed a significant difference in the same direction, *t*(14) = 6.39, *p* < 0.001, 0.70 ± 0.07 vs. 0.58 ± 0.04, Cohen's *d* = 1.65. This indicates that variance in human responses that could not be explained by human-to-human correlations or our model predictions is not just due to noise but also to idiosyncratic behavior.

### Network

The full architecture of the network is shown in Figure 3a. There are four tiers of convolutional processing. The first three of these consist of seven parallel layers of eight filters each that perform convolutional processing at different scales of the image ranging from 1/1 scale to 1/2^6^. In between these are a number of parallel scaling layers, such that every one of the parallel convolutional layers receives the output from all processing scales as input. The fourth tier consists only of one convolutional layer of eight filters that processes the image at full scale. We compared predictions by our full network as well as our threshold model and ground-truth values to human responses (see Figure 5). The full network after training correlated higher than the threshold model or ground truth to the mean of human observers on the target set (*r* = 0.78) and on the validation set (*r* = 0.69).

### Pruning

We then sought to improve further the fit of the network to human performance through pruning. We searched for pruned configurations of our network that responded more similarly to humans using a genetic algorithm. The genetic algorithm searched through configurations (different combinations of on/off settings) for the connections between the first and second and the second and third tiers, shown in blue in Figure 3a.

**Figure 6.**
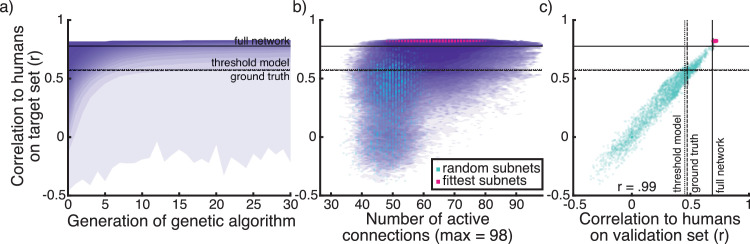
(a) Distribution of correlations to human responses on the target set in each generation, summed across 300 runs of the genetic algorithm. Correlations between humans and ground truth, threshold model and the full network are plotted as lines. (b) Distribution of correlations to human responses on the target set plotted against the number of connections active in each subnet. Plotted in green and pink are 10 random and the 10 fittest subnets from each run of the genetic algorithm (3000 in total each) that are used in (c) and other subsequent analyses. (c) Correlations of a subset of 3000 random and 3000 fittest subnets to human responses on the target set and on the validation set. Correlation between the fit on these two datasets is *r* = 0.99

The results of pruning in terms of correlation with humans on the target pixel set are shown in Figure 6a. Although many pruning configurations reduced correlation to humans, in every run the genetic algorithm discovered pruned configurations of the network that responded more like humans than the full network. The highest correlation between a pruned version of the network and humans was *r* = 0.83, at the upper limit of human-to-human correlations that we observed. Figure 6b shows similarity of pruned configurations to humans for all runs plotted against the number of active connections. Interestingly, there is a large range in the number of connections that are active in configurations that improve the correlation of the network with humans.

### Validation performance

To validate whether pruning according to our target dataset yields generalizable results, we compared responses from a subset of the configurations against humans on the validation dataset. We picked the 10 fittest configurations from the last generation and 10 random configurations from the initial generation from each run of the genetic algorithm, resulting in 3000 fit and 3000 random configurations in total (shown in Figure 6b). The validation performance of these pruned versions of the network can be seen in Figure 6c, along with the validation fitness of ground truth, the threshold model, and the full network (*r* = 0.51, *r* = 0.49, and *r* = 0.69, respectively). The maximum correlation of a pruned network to humans on the validation set we observed was *r* = 0.73. Correlations of the pruned networks with humans on both datasets correlate at *r* = 0.99. The purpose of this analysis is not to test how well the pruned networks match human performance but rather how well this similarity generalizes to other human data. We included random and fit pruning configurations to test this generalizability for configurations that show both higher and lower similarity to humans compared with the full network. The results indicate that the component of human performance that we capture is highly generalizable and not limited to images in the target set. It also indicates that improvement in correlations to humans on the target set suggest a robust shift in network behavior toward more human-like responses. Of the 3000 fit configurations, all correlated with humans higher than the full network on the target set, and 21 did not improve on the correlation of the full network with humans on the validation set.

### Example pruned network

For the following analysis we picked a candidate pruned configuration of our network. Our choice was based on four criteria: (a) variance in human observer data explained (*R*^2^), (b) variance in ground-truth data explained (*R*^2^), (c) variance in human errors explained by network errors (*R*^2^ between the differences of prediction and ground truth and human responses and ground truth), and (d) lowest root mean square error (RMSE) to human observers. We rank-ordered the 3000 fit network combinations used previously in the validation analysis according to each criterion and picked the configuration that showed the lowest sum of ranks. The predictors were all highly intercorrelated (all *r* = 0.82 or greater). We chose to use several selection criteria to avoid possible outliers, such as configurations that correlate well with humans but show very weak responses close to 0 (high correlation but high RMSE to humans). The network we picked according to the four criteria was also the third fittest in terms of *R*^2^ to humans on the target set.

### Differences from full network

**Figure 7.**
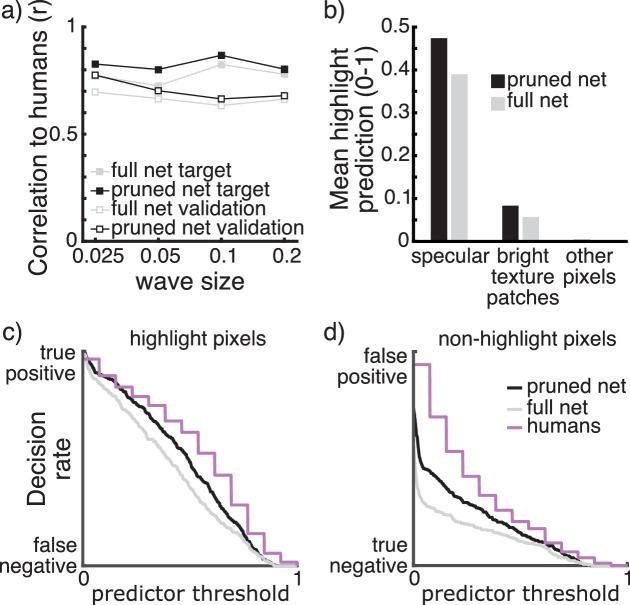
(a) Correlation to humans of the full (gray) and pruned (black) network on the target and validation datasets (filled and empty squares, respectively) seperated for spatial scales of surface geometry. (b) Mean predictions for all pixels in 15,000 images separated into pixels that contain a highlight, bright texture patches, or neither. (c) True-positive and false-negative decision rates for highlight pixels from the target dataset for the full network, pruned network, and human observers if different predictor values act as a threshold in a binary decision. (d) False-positive and true-negative decision rates for non-highlight pixels from the target dataset for the full network, pruned network, and human observers if different predictor values act as a threshold in a binary decision.

To better understand what the pruned network does differently than the full network, we compared network responses to human responses in more detail. We looked at the responses of both networks to different spatial scales of surfaces. The correlation to human observers separated for surface perturbation size (Figure 7a) shows that the pruned network has a tendency to better predict human responses than the full network for all surface scale conditions. This improvement shows in the target and validation datasets. As a test of statistical significance, we calculated the 95% confidence intervals for the difference between each pair of correlations (*r* between the pruned net and humans – *r* between the full net and humans) as suggested by Zou (2007). For the subsets per surface scale of the target dataset, these confidence intervals were (0.03–0.09), (0.05–0.12), (0.02–0.08), and (–0.01 to 0.06), respectively from smallest to largest perturbations (left to right in Figure 7a). For the subsets of the validation dataset, the 95% confidence intervals were (0.05–0.12), (0.01–0.07), (0.00–0.07), and (–0.03 to 0.07), respectively. In the target dataset the pruned net correlated significantly higher than the full net with humans for all perturbation categories, except for the image category with largest perturbations. In the validation dataset, the pruned net correlated significantly higher than the full net with humans for the two image categories with smallest surface perturbations. Where correlations were not significantly different, the observed direction of the difference was also that the pruned net correlated higher with humans than the full net. This indicates that the improvement of the pruned network is larger for images of surfaces with smaller perturbations but not limited to specific spatial scales and includes a mechanism that is applicable over a wide range of geometries.

In another step we looked at the responses of the networks to different categories of pixels to test how the pruning affected sensitivity to highlights (i.e., to answer the question whether pruning elicited a criterion shift). Specifically, we divided the pixels in every image into three groups: specular, bright texture patches (excluding areas that overlapped with specular), and other pixels. For 15,000 images we summarized the network responses as the mean response per pixel category. This result is shown in Figure 7b. Our pruned network appears to give higher responses than the full network to pixels of all categories, but to different degrees. The largest increase in mean glossiness rating is for pixels containing a highlight with a lesser increase for texture pixels and a very small increase for pixels that contain neither highlights nor bright texture patches. In other words, the pruned network seems to make a criterion shift compared with the full network that raises the responses to correctly identified highlights but also (to a lesser degree) to bright texture pixels.

This seems to suggest that the pruned network makes more or stronger true-positive decisions at the cost of an increased false-positive rate. Because the predictors do not make binary decisions but rather give continuous predictions, we visualize this as the rate of true-positive and false-positive decisions if a certain predictor value served as the threshold for binary decisions. Figures 7c and 7d show network predictions of the 720 pixels in the target dataset in this way, compared with mean human ratings. The pruned network does indeed favor true-positive but also false-positive decisions compared with the full network. In doing so, the pruned network shows more similar behavior to the mean of human observers, where this tendency appears to be even more pronounced.

### Network predictions for stimuli with modified highlights

To further investigate similarities between the pruned network and humans, we constructed a set of images containing highlights that we manipulated in the image space. Specifically, we altered the *global rotation* of the entire specular component of the images in angles of 0°, 90°, 180°, and 270°. Anderson and Kim (2009) showed that surfaces with displaced highlights are less likely to be perceived as glossy than when highlights are in their correct positions. These test images contained no texture. We fed the same 1000 images with every rotation condition through our pruned network and compared responses to the (manipulated) highlights contained in the images. We used the RMSE per image between the network predictions and the highlight component (correctly or wrongly oriented) of the images as a measure of how much the network responses match the highlights. Lower RMSE values indicate a stronger tendency to recognize the manipulated highlights as highlights.

**Figure 8.**
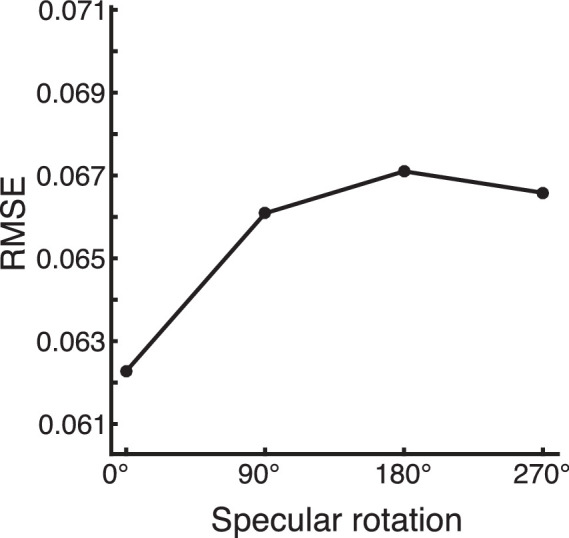
RMSE between predictions by the pruned network and the ground truth specular component for 1000 untextured images with differently rotated specular components. A specular rotation of 0° indicates the correct alignment.

The RMSE between pruned network predictions and rotated highlight components is shown in Figure 8. Global rotations affect the RMSE, but to a very small degree. The original, non-rotated highlights produce the lowest RMSE, indicating that globally rotated highlights are less likely identified as highlights by the network. It is important to note that, although the direction of this effect is in line with what we expect from a human-like model, it is also very small. For comparison, the RMSE between 1000 pairs of random noise images of the same size as our stimuli is 0.412 (*SD* = 0.007). This means that most of the manipulated highlights are still mostly recognized as highlights, only slightly less than the original highlights. Interestingly, this seems to indicate that a highlight detection model can behave much like a human observer in conventional images, without being very sensitive to photo-geometrical constraints.

### Learned representations

To investigate what kinds of representation the pruned network has learned, we compared the responses of units to a number of hand-engineered image and surface descriptors (predictors) in a RSA (Kriegeskorte et al., 2008). We calculated representational dissimilarity matrices (RDMs) consisting of the pairwise Euclidean distances between pairs of images as they are represented in the output of every convolutional neuron throughout the pruned network, as well as by the different predictors. RDMs were calculated based on 4500 images, including all of the texture conditions we used in the training set. We included 34 possible predictors ranging from image-computable local filters and summary statistics to more complex geometrical and intrinsic image (Barrow & Tenenbaum, 1978) components. We grouped the predictors into seven categories (for detailed descriptions, see the Supplementary Material):

1. *Input Image—*original grayscale input image
2. *Summary statistics*—mean, *SD*, skewness, and kurtosis of image intensity
3. *Edge detection/direction*—pixel gradients in *x*- and *y*-directions, local contrast, locally normalized image
4. *Image gradients/anisotropy*—orientation of the smoothed image gradient in *x* and *y*, anisotropy of the smoothed image
5. *Geometry information—*camera distance, angle to camera, light distance, angle to light source, convexity, pointiness (magnitude of local curvature, as calculated by Blender), *x* normal, *y* normal, *z* normal, occluding edges, distance from occluding edges
6. *Intrinsic components—*texture, matte (shading), specular, specular direct, specular indirect, specular coverage, texture coverage
7. *Scene information—*surface scale, texture type, texture condition, scene

**Figure 9.**
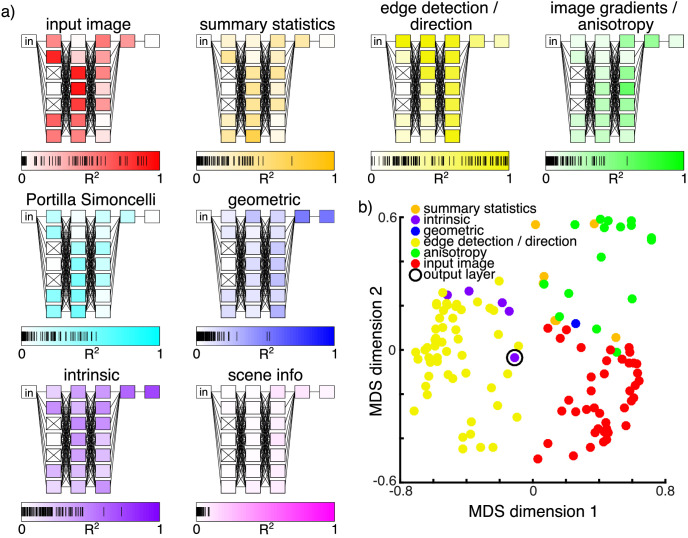
(a) Representational similarities between single filters of the pruned network and predictor categories. Each box represents one convolutional layer with eight filters, and each column of boxes represents seven parallel layers. For a more detailed description of the architecture, see Figure 3 and Methods. The color of each box shows the maximum variance explained for any filter's first-order RDM (upper triangle) per layer (out of eight each) by all predictors in a given category. Layers marked with an × contain only “dead” filters. Markings on the scales below each network schematic show the variance explained (*R*^2^) of all network filter representations by the predictors in the category. (b) The first two dimensions of an MDS representation of all active filters in the pruned network. MDS is calculated on the pairwise similarities between first-order RDMs of representations of 4500 images by individual filters (upper triangle). Each filter is shown as a dot colored according to the category of the most similar single predictor.

We correlated the top triangles of unit and predictor RDMs. Figure 9a gives an overview of where in our pruned network the internal representations show greatest similarities to each predictor category in terms of maximal total variance explained. Every layer contains eight filters; shown in Figure 9a is the maximum per layer that any of these filters is explained by all predictors in a given category. Note that boxes in the figure represent layers, and columns of boxes represent parallel layers that receive the same input at different scales (see Methods). In addition to the predictors mentioned above, we compared network representations with representations according to the texture analysis model by Portilla and Simoncelli (2000), also shown in Figure 9a. This is another hand-engineered model of mid-level vision that has very successfully been used in texture analysis and synthesis.

The results of the RSA broadly agree with the expectation that later network stages are associated with more complex representations. Simpler, directly image-computable predictors tend to show similarities earlier in the network, becoming less relevant toward the output. Surprisingly, however, we see that filters in deeper intermediate layers also show similarities to edge detection and gradient predictors. Similarities to more complex predictors such as geometric or intrinsic parameters emerge only late in the network. Summary predictors show greatest similarities to units that process lower resolution, more spatially summarized representations of the image. The low similarity to predictors from the *scene information* category means that our network has likely not learned the categorical factors by which we constructed the dataset. Portilla–Simoncelli statistics are most similar to units in the first and second tier, especially those operating on spatially summarized representations. This indicates that our model indeed makes use of features similar to hand-engineered mid-level features. The placement of similar neurons in the network suggests that these features occur at intermediate processing stages, with other features in later layers computed based on them.

Figure 9b shows an arrangement of the individual units of the pruned network according to the similarity of their response characteristics. Specifically, we used the RDMs of individual network units (described above) to compute a second-order RDM. This second-order RDM contained the pairwise dissimilarities between individual network units (calculated as the correlation coefficients of the upper triangles of first-order RDMs subtracted from 1). We then visualized the similarities in two dimensions using multidimensional scaling (MDS). Each point represents a single unit, colored according to the categories of their single most similar predictors. This reveals broad clusters of qualitatively similar units. Most filters show the greatest similarities to the original input image or to image-computable predictors from the categories *edge detection/direction* and *image gradients/anisotropy*. A small number of filters (the network output among them) are most similar to geometric predictors or to intrinsic image components. In the case of the output layer, this presumably reflects the objective function, which was to return a per-pixel highlight map (i.e., an intrinsic image of the specular component). Thus, as expected, units that correlate with relatively high-level factors such as surface geometry or intrinsic images tend to be more prevalent in later stages of the network.

### Lesion analysis

Having broadly classified the functions of individual neurons, to assess their relative importance for to the network's overall functionality we performed a lesion analysis. We lesioned every neuron in the pruned network individually by setting all weights of the respective neuron to 0. For every neuron we tested the lesioned network in terms of loss on the validation image set (15,000 images) and correlation to humans on the target set we used earlier (720 individual pixels). It is important to note that this analysis is different from the pruning used to identify network variations that more closely matched humans, where we pruned outgoing connections of neurons (of which there are several for each neuron in the first two tiers).

We correlated both lesion scores with the variance of representations of each neuron explained by representations in each category of predictors (summarized in the bars under each network plot in Figure 9a). We find no substantial proportion of variance in loss or similarity to humans due to lesioning explained by the similarity of the lesioned neurons to any predictor category (the largest being *R*^2^ = 0.03 between the similarity of individual neurons to edge detection/direction predictors and loss of the network with the respective neuron lesioned). The importance of neurons to network performance cannot be explained by similarity to any of the predictor categories. Put differently, the function of the network as a whole seemingly depends on all of the different classes of function roughly equally. This can be contrasted with previous findings, where particular classes of node were of special importance to overall network function (van Assen, Nishida, & Fleming, 2020).

## Discussion

We trained a neural network to identify specular highlights in computer renderings of surfaces. This is a challenging mid-level visual inference about the causal origin of bright patches in images, which is considered a key stage in the perception of gloss (Beck & Prazdny, 1981; Berzhanskaya, Swaminathan, Beck, & Mingolla, 2005; Fleming, 2012; Kim et al., 2011; Todd et al., 2004). Unlike other recent work using deep learning to identify highlights in the machine vision literature (Attard, Debono, Valentino, & di Castro, 2020; Fu, Zhang, Lin, Zhu, & Xiao, 2020; Lin, el Amine Seddik, Tamaazousti, Tamaazousti, & Bartoli, 2019; Madessa, Dong, Gan, & Gao, 2020), our focus was on matching human performance rather than achieving the best possible accuracy from an engineering perspective. To do this, we trained a neural network to identify highlights and used pruning to identify a subnetwork within the trained network that responds more like human observers than the full network. We found not one but many configurations that do this (Figure 6a). The best of these significantly outperformed both a simple threshold operation and ground truth at predicting human judgments. To our knowledge, this is the first image-computable model that predicts average human highlight perception judgments at approximately the same level as individual participants do.

An important limitation of this study is that it only concerns rendered stimuli under a very simple lighting environment. We chose to use renderings because this gave us access to ground truth, which would not be possible with natural images. Although a number of approaches exist for approximating the specular component, such as other image decomposition models or polarizing filters, to the best of our knowledge none of these provides such a good estimate of the ground truth as rendering. Our reason for choosing a simple illumination environment was to ensure that it is clearly defined whether or not a pixel contains a highlight, which was necessary for our experiment with human observers. It is reasonable to consider only specular reflections of light sources (direct illumination) as highlights, as reflections of other surfaces (indirect illumination) are usually significantly dimmer. Yet, under natural lighting (e.g., with a light probe illumination), light sources usually occupy only a small proportion of incoming directions. Most incident directions deliver light from secondary sources. There is no simple way to separate direct from indirect illuminations with complex natural illumination patterns, so we used a more controlled approach with simple artificial light sources, allowing highlights to be formally defined and identified. In a natural image, every pixel would contain some component of specular reflection. Due to this difference in specular distribution throughout the images we would expect our model to perform poorly on photographs of natural scenes. Several studies on the human perception of gloss have focused on highlights as distinct visual elements, which has been a successful approach for modeling human gloss responses (e.g., Fleming, 2012; Marlow et al., 2012). An important question along the way to generalizing this present study to natural images is to what extent humans perceive isolated specular highlights or continuous specular images in natural scenes.

Our network consists of 61,505 trainable parameters and is designed to respond to 65,536 pixels for one individual image. Importantly, we find that the similarity of pruned networks to humans is highly consistent on both target and validation datasets, correlating at *r* = 0.99 for a subset of 3000 random and 3000 fit pruning configurations. A small dataset of 720 individual pixels was sufficient to identify a component of network responses that transfers to another independent dataset with data from different observers responding to different stimuli. This indicates strongly that the component in the network that was emphasized by pruning is robust across random variations in our dataset. This opens up other possible applications for pruning where target data are difficult to come by and a small target dataset could be used to fine-tune a model that is pretrained on simulated data.

We found configurations that correlate better with humans than the full network in which more than half of all connections were pruned. This can have several possible explanations. One is that there are superfluous connections that make little or no impact on the behavior of the network overall or do not influence its similarity to humans. Another possibility is that connections are redundant. A third explanation could be that there are many alternative routes that the network can take to act more human. It seems likely that superfluous connections and neurons play at least a part in this, due to the high number of dead neurons (55 out of 177 in the pruned network) that we have seen in later analysis. In the RSA we also saw that a large number of neurons show a high similarity in their representations to image-computable features. This high similarity to the same predictor or groups of predictors also makes the possibility of alternative routes within the network seem plausible.

Picking one representative pruned configuration, we summarized the different responses of the pruned and the full network in terms of mean predictions of pixels belonging to different image regions (specular patches, bright texture patches, and all other pixels). The pruned network shows a higher prediction mean for all categories (i.e., it is more likely to recognize them as highlights), but the increase is largest for specular pixels, making the difference between overall predictor mean for specular and texture pixels larger than it is for the full network. Put another way, the pruned network acts as if it has a (liberal) criterion shift relative to the full network, accepting more highlights than the original. Although it might be tempting to think that the pruned network performs better in terms of the original training criterion of identifying highlights, this is not the case. The mean per-pixel predictions in Figure 7b ignore the number of pixels in each category whereas the training loss does not. This criterion shift opens up questions about whether human perceptual decisions (especially in ambiguous cases) are driven by maximizing true positives or correct reject decisions, and indeed our results indicate that humans are more likely than the full or pruned networks to make true-positive decisions, at the same time also increasing the rate of false positives compared with our networks. Perhaps false-positive decisions in recognizing highlights are not as important to humans, and it is questionable whether the exact area covered by highlights plays a similar role to humans in this context as it does to our networks as defined by the training loss. If a measurement related to coverage does play a role in human decisions, it seems likely that highlights weigh more than their actual coverage, given that highlights propagate the impression of glossiness to a larger image area (Berzhanskaya et al., 2005), but this requires further investigation.

Previous studies have emphasized the role of so-called photo-geometric constraints in identifying and interpreting highlights (Anderson & Kim, 2009; Beck & Prazdny, 1981; Kim et al., 2011; Marlow et al., 2011; Todd et al., 2004). In order for a bright image patch to be a specular reflection, it must align in orientation and position with the underling surface geometry and/or shading patterns. We do find that global rotation of the highlight component in our stimuli leads to a slight increase in prediction error (RMSE) by our pruned network compared with the manipulated specular map. This indicates that the network has learned to a limited degree to use orientations and positions of patches as a cue to identify highlights; however, the magnitude of the effect is small, suggesting that these constraints do not weigh heavily. Although it shows some sensitivity to these constraints, our model still largely characterizes misaligned highlights as highlights, failing to replicate human behavior under such conditions as reported by Anderson and Kim (2009) and Marlow et al. (2011). It is interesting to note that a model can predict a large amount of variance in the human data without being very sensitive to violations of these constraints. This is similar to the results of Prokott, Tamura, and Fleming (2021) where we found that neural networks trained to discriminate high- from low-gloss materials show little difference in their response when presented with images with displaced specular components.

Similar to these globally rotated highlights, our training and test images included conditions with false highlights, where we applied highlights from a different scene as texture. We included these as a challenging condition similar to stimuli used in Anderson and Kim (2009), but with both correctly and incorrectly placed highlights in one image. As with rotated highlights, our network erroneously identified most of these false highlights as highlights. Although this is underperformance in terms of the training objective, our network is a better predictor of human responses to these images than the threshold model or ground truth, and the pruned network predicts human responses even better for the target set. Correlations to humans on pixels from these images in the target set are *r* = 0.66, *r* = 0.27, *r* = 0.76, and *r* = 0.78 for threshold model, ground truth, full network, and pruned network, respectively (validation set *r* = 0.65, *r* = 0.24, *r* = 0.74, and *r* = 0.73, respectively). Although Anderson and Kim (2009) and Kim et al. (2011) showed that surfaces with displaced highlights are less likely to be perceived as glossy, we find some evidence that false highlights are rather equally perceived as highlights. We also find that the threshold model relying only on image intensity is a better predictor for human responses to these stimuli than for other texture conditions. However, it should be noted that this is not the focus of this study and that this subset of stimuli consisted of only 96 individual pixels.

In an RSA, we find similarities between the representation at single neurons in the network to various candidate predictors. Generally, we find that similarity to low-complexity predictors that are computable directly from the image occurs throughout the network but also earlier than more complex predictors. Complex geometric and intrinsic predictors show similarities only to very late neurons. We find similarities to summary predictors in spatially summarized units of the network. An alternative way of looking at the data is to classify each neuron according to the category of its most similar predictor (Figure 9b). This reveals that a very large proportion of neurons is most similar to either the input image or an image-computable predictor describing either edges and pixel contrast or the image gradients and anisotropy. However, in a lesion analysis, the similarity of a neuron to any category of predictors was not predictive of its impact (when lesioned) on the model loss or its similarity to humans. Our data do not attest particular importance to any predictor category. This suggests that neurons similar to various predictors are important for the network to perform well and to predict human highlight perception.

A possible way of interpreting these results is that humans use different strategies. Where there are conflicting cues such as congruent and incongruent highlight-like patches, it might be that humans resort to simpler, less conflicting cues, such as luminance. Human responses to stimuli containing false highlights as textures are more similar to predictions by the threshold model than human responses are overall. This represents one case in which humans responded very much like a simple intensity-based model. Our results suggest that photo-geometrical constraints are not the single most important cue to human gloss and highlight perception and will not prevail over simpler factors such as overall relative brightness under all conditions.

## Conclusion

We investigated human perception of highlights on glossy surfaces that also contain different types of bright texture patches. We developed, to our knowledge, the first image-computable highlight-detection algorithm that reproduces many of the successes and failures of human judgments (with the notable exception of effects of highlight orientation). We demonstrated an application of pruning using a genetic algorithm as a method for fine-tuning a neural network trained on simulated physical data to a sparse dataset of human responses. Improvements due to pruning in network similarity to human judgments on a target dataset transfer well to a parallel dataset and are consistent over different stimulus conditions. On both datasets, the pruned networks correlate with humans better than the full network and as high as the maximum human-to-human correlation we observed. Compared with the unpruned network, a pruned example network shows a criterion shift that makes false-positive judgments slightly more likely while at the same time increasing the average difference in responses between pixels that contain a highlight and pixels that do not. We see modest evidence that our network has learned to use photo-geometric cues to identify whether bright patches are highlights, but these effects are very small. In an RSA and subsequent lesion analysis, we find no evidence that neurons that are similar to geometric predictors (or any other class of predictors) are especially important for the network to achieve low objective loss or high similarity to humans. The lesion analysis provides no evidence that photo-geometric cues are particularly important for the network to respond similarly to human observers, suggesting that not only these relatively complex computations are being used by the human visual system in perceiving highlights.

## Supplementary Material

Supplement 1
