# Supplementary material for: Identifying specular highlights: Insights from deep learning
Source: J Vis. 2022 Jun 17;22(7):6. doi: 10.1167/jov.22.7.6 (PMC9206496; doi:10.1167/jov.22.7.6)
Supplement: Supplement 1 [file jovi-22-7-6_s001.docx]

**Supplementary Material**

*Detailed descriptions of predictors:*

This is a list of the predictors we used in the representational similarity analysis (RSA; Kriegeskorte, Mur, & Bandettini, 2008). For every predictor (written in bold below) we calculated a separate representational dissimilarity matrix (RDM). RDMs were calculated differently for each predictor, as described below. We grouped the 34 predictors into 7 categories (Input image, summary statistics, Edge detection / direction, Image gradients / anisotropy, geometry information, intrinsic components, and scene information). Note that we placed the input image in a separate category containing only one predictor.

Input image: *The RDM of this predictor was based on the Euclidean distance between the 65536 dimensional vectors of individual images ( = 256 × 256 pixels; the full image size)*

*The* ***input image*** in one channel grayscale

Summary statistics: *Each of these predictors describes an image with a scalar. The RDMs of these predictors were each based on the absolute differences between these scalars.*

***mean intensity*** per image

***standard deviation*** ***of intensity*** per image

***skewness of intensity*** distributions per image

***kurtosis of intensity*** distributions per image

Edge detection / direction: *The RDMs of these predictors were each based on the Euclidean distances between 65536 dimensional vectors of individual images*

***pixel gradients in x direction*** - using a sobel function

***pixel gradients in y*** ***direction*** - using a sobel function

***local contrast*** – the variance in each 3x3 pixel patch

***locally normalized image*** – the difference between an individual pixel and the mean of the 3x3 pixel patch of which it is the center

Image gradients / anisotropy: *The RDMs of these predictors were each based on the Euclidean distances between 65536 dimensional vectors of individual images*

These predictors are based on the structure tensors of the images (Knutsson, 1989), which we calculated from image gradients smoothed using a gaussian with sd = 5. The structure tensor again was smoothed with a gaussian with sd = 5.

***gradients of the smoothed image in x direction*** – the x component of local image orientation as described by the first component of the second eigenvector of the structure tensor

***gradients of the smoothed image in y direction*** – the y component of local image orientation as described by the second component of the second eigenvector of the structure tensor

***anisotropy of the smoothed image*** – the ‘coherence’ of the image calculated locally as $c=\left( \frac{\mu_{1}-\mu_{2}}{\mu_{1}+\mu_{2}} \right)^{2}$; where μ_1_ and μ_2_ are the first and second eigenvalues of the structure tensor respectively (Jähne, 1993)

Geometry information: *The RDMs of these predictors were each based on the Euclidean distances between 65536 dimensional vectors of individual images*

***camera distance*** – distance between the camera and the surface at each pixel in the image. The units are arbitrary but standardized for all images

***angle to camera*** – the angle between the surface normal at each pixel and the camera

***light distance*** – the distance between the surface at each pixel and the center of the light source

***angle to light source*** – the angle between the surface normal at each pixel and the direction to the center of the light source

***convexity*** – the mesh curvature per vertex at each pixel in the image. This was obtained from the rendering scenes in Blender using the ‘pointiness’ property of the geometry node.

***pointiness*** –the unsigned difference of the convexity data to zero curvature

***x normal*** – the x component of the normal vector of the surface at each pixel

***y normal*** – the y component of the normal vector of the surface at each pixel

***z normal*** – the z component of the normal vector of the surface at each pixel

***occluding edges*** – marking the location of edges that occlude other parts of the surface. These were rendered with Blender’s ‘Freestyle Line Set’ functionality, using the ‘Silhouette’ edge type. These lines mark the edges between regions of a surface that face towards or away from the camera.

***distance from occluding edges*** – distance in image space of each pixel from the nearest occluding edge

Intrinsic components: *The RDMs of the predictors ‘texture’, ‘matte’, ‘specular’, ‘specular direct’ and ‘specular indirect’ were each based on the Euclidean distances between 65536 dimensional vectors of individual images. The RDMs of ‘specular coverage’ and ‘texture coverage’ were each based on absolute differences between scalars for each image.*

***texture*** – the (grayscale) texture component of each image (without shading information)

***matte (shading)*** – the diffuse component of each image

***specular*** – the ground truth specular reflections for each image

***specular direct*** – only direct specular reflections of the light source

***specular indirect*** – all indirect specular reflections (interreflections) for each image

***specular coverage*** – the proportion of pixels covered by specular reflections in each image

***texture coverage*** – the proportion of pixels covered by bright texture markings in each image

Scene information: *Details on how the RDMs were calculated are given in the description of each predictor.*

***surface scale*** – the scale of the geometry of the surface. The surfaces for the scenes were created using Blender’s ‘ocean’ simulation, the scale was determined by the ‘smallest wave’ parameter. We used 4 values spaced equally on a log2 scale. For the predictor RDM we used an ordinal scale – the RDM indicates how many steps apart the surface scale of two images is.

***texture type*** – the category of texture of each image – marble, Voronoi, checkered, untextured or false highlights. This factor was used nominally so the RDM was binary, indicating whether the texture type for two images was the same or different

***texture condition*** – the category and scale of texture of each image. For Voronoi, marble and checker textures there are 4 texture scales, plus two different false highlight and the untextured conditions making a total of 15 texture conditions for each scene. The RDM is binary, showing whether two images are of the same texture condition or not.

***scene*** – a nominal factor resulting in a binary RDM that showed whether two images shared the same underlying scene / geometry.

Supplementary material references:

Jähne, B. (1993). *Spatio-Temporal Image Processing: Theory and Scientific Applications* (1st ed.). Springer-Verlag. https://doi.org/https://doi.org/10.1007/3-540-57418-2

Knutsson, H. (1989). Representing local structure using tensors. *Proceedings of 6th Scandinavian Conference on Image Analysis*, 19–22. https://doi.org/10.1007/978-3-642-21227-7_51

Kriegeskorte, N., Mur, M., & Bandettini, P. (2008). Representational similarity analysis - connecting the branches of systems neuroscience. *Frontiers in Systems Neuroscience*, *2*, 4. https://doi.org/10.3389/neuro.06.004.2008
